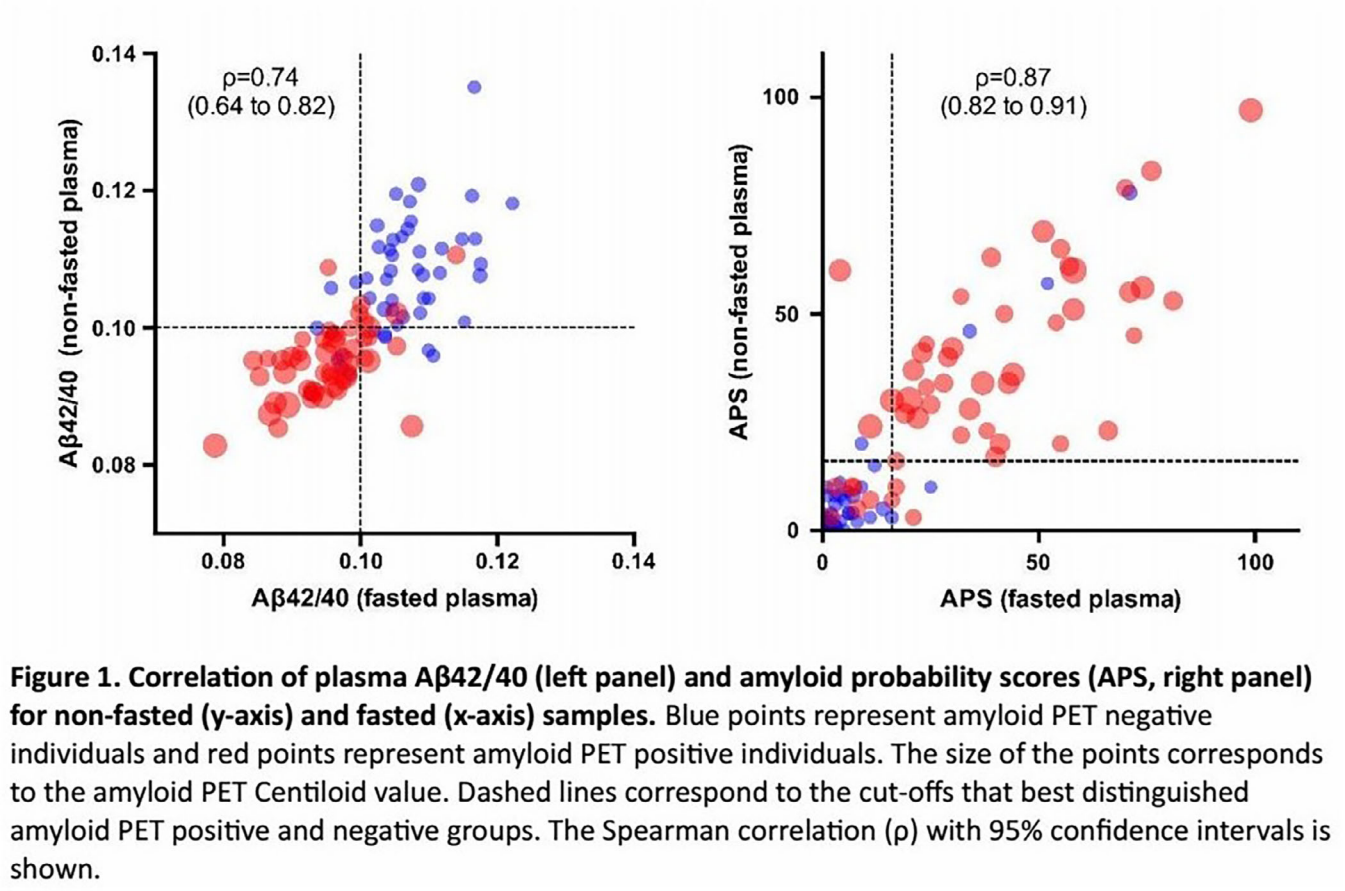# Consistent classification of amyloid status by fasted and non‐fasted plasma Aβ42/40 and Amyloid Probability Score

**DOI:** 10.1002/alz.091937

**Published:** 2025-01-09

**Authors:** Samuele Bonomi, Tammie L.S. Benzinger, Chengjie Xiong, John C. Morris, Joel B. Braunstein, Suzanne E. Schindler

**Affiliations:** ^1^ Washington University School of Medicine in St. Louis, St. Louis, MO USA; ^2^ Hope Center for Neurological Disorders, Washington University in St. Louis, St. Louis, MO USA; ^3^ Mallinckrodt Institute of Radiology, Washington University in St. Louis, St. Louis, MO USA; ^4^ Knight Alzheimer Disease Research Center, St. Louis, MO USA; ^5^ C2N Diagnostics, LLC, Saint Louis, MO USA; ^6^ Washington University in St. Louis School of Medicine, St. Louis, MO USA

## Abstract

**Background:**

It is unknown whether fasting status affects classification of amyloid status by plasma Aβ42/40.

**Methods:**

The cohort included 50 amyloid PET positive and 50 amyloid PET negative individuals enrolled in studies of memory and aging at the Knight Alzheimer Disease Research Center (ADRC). Included individuals had a non‐fasted plasma sample and an amyloid PET scan performed within 3 months of a fasted plasma sample. Non‐fasted samples were collected throughout the day. CSF was collected at approximately 8 am at the same session as the fasted plasma sample. Plasma Aβ42/40 was measured by an immunoprecipitation‐mass spectrometry (IPMS) assay at C2N Diagnostics. The classification accuracy of amyloid PET and CSF status was evaluated in the fasted and non‐fasted samples by logistic regression. Cut‐offs for amyloid positivity with the highest Youden index (combined sensitivity and specificity) were selected. The correlations of fasted and non‐fasted plasma Aβ42/40 and amyloid probability score (APS, a modeled value incorporating plasma Ab42/40, ApoE proteotype, and age) were evaluated.

**Results:**

Participants had an average age of 69.3 ± 8.3 years (mean ± standard deviation) and 94 of 100 were cognitively unimpaired. The average interval between collection of fasted and non‐fasted plasma samples was 1.8 ± 0.7 months. Classification accuracy of amyloid status based on fasted or non‐fasted samples (either plasma Aβ42/Aβ40 or APS) was almost identical: for amyloid PET status, ROC AUC 0.88 and 0.89 respectively; for CSF Ab42/40 status, ROC AUC 0.89 and 0.91 respectively. The APS cut‐off for amyloid PET status and CSF status was 16 for both fasted and non‐fasted samples; based on this cut‐off, 8 of 100 samples had discordance between fasted and non‐fasted plasma APS. The Spearman correlation between fasted and non‐fasted plasma for Ab42/40 was ρ=0.74 (0.64‐0.82) and for APS was ρ=0.87 (0.82‐0.91).

**Conclusions:**

Measurements of plasma Aβ42/40 with an IPMS assay and the APS had high and consistent associations with amyloid PET and CSF Aβ42/40 status in fasted and non‐fasted samples. These results suggest that fasting status, and potentially the time of day that a sample is collected, does not significantly affect classification of amyloid status by plasma Aβ42/40 or APS.